# Coronary Atherosclerotic Plaque Detected by Computed Tomographic Angiography in Subjects with Diabetes Compared to Those without Diabetes

**DOI:** 10.1371/journal.pone.0143187

**Published:** 2015-11-23

**Authors:** Bahram Khazai, Yanting Luo, Steven Rosenberg, James Wingrove, Matthew J Budoff

**Affiliations:** 1 Department of Cardiology, University of Miami Miller School of Medicine, Miami, Florida, United States of America; 2 Department of Cardiology, Los Angeles Biomedical Research Institute at Harbor-UCLA, UCLA School of Medicine, Torrance, California, United States of America; 3 CardioDx, Inc., Palo Alto, California, United States of America; Baker IDI Heart and Diabetes Institute, AUSTRALIA

## Abstract

**Objectives:**

Little data are available regarding coronary plaque composition and semi-quantitative scores in individuals with diabetes; the extent to which diabetes may affect the presence and extent of Coronary Artery Calcium (CAC) needs more evaluation. Considering that this information may be of great value in formulating preventive interventions in this population, we compared these findings in individuals with diabetes to those without.

**Methods:**

Multi-Detector Computed Tomographic (MDCT) images of 861 consecutive patients with diabetes who were referred to Los Angeles Biomedical Research Institute from January 2000 to September 2012, were evaluated using a 15–coronary segment model. All 861 patients underwent calcium scoring and from these; 389 had coronary CT angiography (CTA). CAC score was compared to 861 age, sex and ethnicity matched controls without diabetes after adjustment for Body Mass Index (BMI), family history of coronary artery disease, hyperlipidemia, hypertension and smoking. Segment Involvement Score (SIS; the total number of segments with any plaque), Segment Stenosis Score (SSS; the sum of maximal stenosis score per segment), Total Plaque Score (TPS; the sum of the plaque amount per segment) and plaque compositionwere compared to 389 age, sex and ethnicity matched controls without diabetes after adjustment for BMI, family history of coronary artery disease, hyperlipidemia, hypertension and smoking.

**Results:**

Diabetes was positively correlated to the presence and extent of CAC (P<0.0001 for both). SIS, SSS and TPS were significantly higher in those with diabetes (P<0.0001). Number of mixed and calcified plaques were significantly higher in those with diabetes (P = 0.018 and P<0.001 respectively) but there was no significant difference in the number of non-calcified plaques between the two groups (P = 0.398).

**Conclusions:**

Patients with diabetes have higher CAC and semi-quantitative coronary plaque scores compared to the age, gender and ethnicity matched controls without diabetes after adjustment for cardiovascular risk factors. Since mixed plaque is associated with worse long-term clinical outcomes, these findings support more aggressive preventive measures in this population.

## Introduction

Diabetes Mellitus (DM)is a major risk factor for the earlydevelopment of coronary artery disease (CAD), and people with type 2 DM (DM2) present with CAD occurring at a much younger age comparedto non-DM counterparts [1]. Even patients with DM2 without symptoms of CAD mayhave significant coronary artery calcium (CAC) [2]. For patients with DM, CAC is an independent correlate of myocardial infarction (MI)[3].

The overall risk for thepresence of CAC is four times greater for a young adult with DM than an age-matched person withoutDM[2, 4]. The risk increases if other comorbidities e.g., hypertension (HTN), dyslipidemia, smoking, andfamily history of CAD are taken into account. These results suggest that many young adults with DM haveclinically silent but potentially serious coronary artery lesions based on radiographic evidence.

InType 1 Diabetes Mellitus (DM1),CAD is the main cause of death, with 35% mortality caused by CAD in DM1 patients by 55 years of age in contrast to 4% of non-DM women and 8% of non-DMmen[5]. The benefits of improved DM care do not appear to have reduced CAD mortality in this population [6]. Traditional risk factors for CAD in DM1 including waist to hip ratio, nephropathy, lipids (both HDL and non-HDL cholesterol), HTN and cigarette smoking are predictors of CAD [7–11], however, not all risk can be accounted for by these factors alone, and therefore, other markers for CAD are under investigation to improve the ability to predict risk and set treatment goals. Haffner et al showed that the risk of mortality from cardiovascular causes in subjects with DM2 and no previous history of cardiovascular events is equivalent to non-DM subjects with prior myocardial infarction (MI) [12].

This increased risk of significant cardiovascular events from diabetes led to guidelines that alladults over the age of 40 with DM be treated according to the criteria reserved for secondaryprevention of CAD [13].

### Use of Coronary Calcium to predict CAD in DM

The use of computed tomography (CT) for assessment of CAChas been described as an effective risk stratification modality in asymptomatic patients, and specifically recommended by the American College of Cardiology Foundation and American Heart Association(ACCF/AHA) for individuals with DM, due to data showing advanced risk stratification [14, 15]. Markers like CAC have been shown to be a predictor of cardiovascular (CV) events in older patients with DM.CAC is considered a marker of atherosclerosis [2, 16] and an independent andincremental marker of increased risk of CV events [17]. Furthermore, there is a tight correlationof CAC score to angiographic disease [16–18].

### CT coronary angiography to evaluate plaque morphology

A more direct evaluation of atherosclerosis than CAC is the measurement of total plaque in the coronary arteries. This is possible with coronary CT angiography (CCTA). There are concerns that young patients with DM will have lower levels of CAC (and often zero scores), but may have increased non-calcified plaque which can be assessed on cardiac CT angiography.

Recently noninvasive CT-based plaque imaging has been shown to identify the presence and extent of non-calcified atherosclerotic plaque, which is a better measure of early and total atherosclerosis. Further, cardiac CT can detect sub-clinical (asymptomatic) CAD and mild non-obstructive coronary stenosis, which enhances its ability to identify individuals at risk before the inception of disease [19]. Cardiac CT is an accurate, non-invasive and inexpensive test that measures not only CAC, but also non-calcified plaque, stenosis severity; occult scar (old infarction) and thoracic plaque by use of a contrast enhanced scan [20]. Results from cardiac CT have been comparable to those measured at autopsy [19].

Multiple dose reduction strategies, including significant hardware and software advances over the last 3 years, now allow CT angiography to be performed with <3 mSv doses of radiation, allowing use of this technique to evaluate asymptomatic persons. The scan takes less than 30 minutes [20], and doses administered are lower than annual background radiation doses.

Considering that there is little data available about plaque burden and plaque morphology (mixed,calcified and non-calcified) in patients with DM, we decided to compare these findings in individuals with DM to those without DM.

## Materials and Methods

All research involving human participants have been approved by our institutions Institutional Review Board (IRB). Written informed consent was obtained from the participants and saved either as a hard copy or an electronic copy in the patients charts.

We evaluated 861 consecutive patients with DM who were referred to Los Angeles Biomedical Research Institute(Torrance, CA) from January 2000 to September 2012. Major indications were rule out cardiac etiology of chest pain or equivocal exercise stress test results.All 861 underwent calcium scoring and from these; 389 underwent CTA. All patients were older than 18 years and all the subjects provided informed consent. For CTA, we excluded those with known allergy toIodine and serum creatinineof >1.4 mg/dL. Those with poor quality images, poor contrast enhancement,and with image artifacts (misalignment, motion, and helicalinterpolation) were excluded from the study.

A 64-slice MDCT scanner (LightSpeed VCT; GE Medical Systems, Milwaukee, WI) was used to obtain CAC and MDCT Image acquisition and post- processingCAC studies.

We used prospective electrocardiographic triggering with a tube voltage of 120 kV and tube currents of 300 to 600 mA on unenhanced scans, and imaged the coronary arteries at a temporal resolution of 227 ms and a slice thickness of 2.5 mm during 75% of the RR interval.

Subjects with a baseline heart rate above 65 beats/ min were given oral Beta-blocker to slow their heart rate. Intravenous metoprolol was given in 5-mg increments to a maximum dose of 40 mg to achieve a resting heart rate <65 beats/min in subjects who did not achieve heart rate of <65 with oral Beta-blocker. Finally we scanned all patients eligible for MDCT imaging, irrespective of achieving the goal heart rate of <65 beats/min.

Sublingual nitroglycerine (0.4 mg) was given prior to contrast injection after obtaining antero-posterior and lateral chest X-ray. 10–20 mL contrast bolus was given to detect optimal time for contrast opacification at a level immediatelysuperior to the ostium of the left main artery in the axial image (the location was derived from the calcium scan).60 mL of iodinated contrast(Omnipaque; GE Medical Systems) was subsequently injected using a triple-phase contrast protocol: 40 mL of contrast, followed by 40 mL of a 40:60 mixture of contrast and saline, followed by flush with 50 mL of saline.

We performed retrospective electrocardiographically gated helical contrast-enhanced MDCT imaging. We initiatedthe scans from 10 mm above the level of the left main artery to 10 mm below the inferior myocardial apex with scan parameters being 64 0.625 mm sections (2.5mm); with collimation tube currents of 350 to 780 mAand tube voltage of 100 or 120 kV.

Dose modulation techniqueswere used in all cases to reduce radiation. Multiphase reconstruction of the MDCT scans was performed with reconstructed images from 70% to 80% in 5% increments and 5% to 95% in 10% increments after completion of the scans. The MDCT images were transferred to a reading center for three-dimensional image analysis (Advantage Workstation; GE Healthcare, Milwaukee, WI) at Los Angeles Biomedical Research Institute at Harbor-UCLA Medical Center (Torrance, CA).

An experienced reader blinded to all patient characteristics interpreted all MDCT images. The MDCT image reader was permitted touse any or all available post-processing image reconstruction algorithms, including two-dimensional axial or threedimensionalmaximal intensity projection, volume-rendered, multi-planar reformat and cross-sectional analysis techniques.

### Measurement of CAC Score

We used noncontrast images for CAC scoring.Presence of CAC in a coronary artery was defined as a densityof >130 Hounsfield units (HU) in three ormore contiguous pixels (>1 mm^2^) overlying that coronaryartery. CAC was quantified by using the Agatstonscoring method which has beenpreviously described[21]. We calculated score of each lesion by multiplyingthe lesion area by the density factor obtained from themaximal HU in that specific area. We considered a density factor of 1 for lesionswith a maximum density of 130 to 199 HU, 2 for a maximumdensity of 200 to 299 HU, 3 for a maximum density of 300 to399 HU, and 4 for density >400 HU. Sum of the individual lesion scores from each offour anatomic sites (left main, left anterior descending,circumflex, and right coronary arteries) was considered as total calcium score.

### Measurement of Plaque Burden

We used the 15-segment AmericanHeart Association classification [22] for assessment of coronary arteries. Coronary artery segmentswith diameters >2 mm measured on MDCT imaging were included in the analysis. Usingaxial, cross-sectional and curved multi-planar reconstruction images, presence or absence of any atherosclerotic plaque was evaluated in each segment of the coronary arteries. Coronary plaques were defined as structures >1 mm^2^ within and/or adjacentto the coronary artery lumen, which could be clearly distinguishedfrom thesurrounding pericardial fat tissue and the contrast-enhanced vessel lumen.

Each coronary segment was assigned one coronary plaque regardless of the numberof lesions in that specific segment. We quantified the amount of plaque in eachsegment as mild (score of 1), moderate (scoreof 2), or severe (score of 3) using a previously describedmethod [23]. We calculated total plaque score (TPS) bysummation of the amount of plaque of each coronary segment(maximum score, 45). The severity of diameterstenosis was assigned a score of 0 for normal (no stenosis), 1for 1% to 49% stenosis, 2 for 50% to 69% stenosis, and 3 for≥70% stenosis for each segment. We calculated segment stenosis score(SSS) as the sum of the maximal stenosis scorein each segment (maximum score, 45).

In those segments which were found to have multiple stenoses, the segment with highestdegree of stenosis was taken for analysis. We determined segment involvement score(SIS) by adding the number of segmentswith lesions (maximum score, 15). We assessed the morphology of coronary artery plaques visually. Calcified plaques were definedas those with the presence of >50% calcifications.

Non-calcified plaques were defined as thosewith no calcifications in the plaque and partially calcified plaques (mixed plaques)were defined as having <50% calcifications (lesionswith density >130 HU).

### Covariates

At the baseline visit, age, gender, race (White, Hispanic, Black, Asian and other), BMI (kg/m^2^), family history of CAD, history of chest pain, hyperlipidemia, HTN and smoking status were recorded. BMI was calculated by dividing weight in kilograms by height in meters squared.Using a Dinamap model Pro 100automated sphygmomanometer (Critikon, Tampa, FL), right arm resting seated BP was measuredtwotimes at 1-minute intervals with average of the two readings used for analysis. HTN was defined as receiving antihypertensive medications or a blood pressure ≥140mm Hg systolic or 90 mm Hg diastolic[24]. DM was defined as receiving hypoglycemicmedications or fasting blood glucose of≥126 mg/dl and dyslipidemia was defined as being on lipid lowering medications or based on criteria reported by ATP III [25].

### Statistical Analysis

The data were first analysed using the Shapiro-Wilk normality test. Continuous variables were presented as mean ± SD or median (quartile 1, quartile 3), and categorical variables were presented as frequency (percentage). For variables with normal distributions, Student *t* test for two groups was used to determine the statistical significance between the diabetic and non-diabetic groups. For the variables failing the normality test, statistical significance between groups was determined by non-parametric Mann-Whitney *U* test. To access the categorical variables, the chi-square test was used. For assessing the qualitative relationship between the CACs and semi-quantitative coronary plaque scores with diabetes, the linear regression analysis was performed. Regression coefficients, 95% confidence interval (95% CI) and p-values were reported to characterize both the strength and (statistical) significance. All statistical significance was defined as p< 0.05 (2-tailed). All analyses were performed using SAS software (version 9.4, SAS Institute, Cary, North Carolina).

## Results and Discussion

Baseline characteristics of all subjectsand those with CTA; are shown in Table 1 and Table 2 respectively. 5.2% of the DM subjects and 4.9% of the controls were 40 years old or younger. The mean age of the total study population (861 patients)was 62.1±12.5 years (63% male). Those with DM had a higher BMI (29.9±7.9 vs 26.0±7.1, P<0.0001) and a higher prevalence of dyslipidemia (76.2% vs 54.3% P<0.0001), HTN (77.9% vs 43% P<0.0001), family history of CAD (66.4% vs 52.6%, P<0.0001) and history of chest pain (36.1% vs 24.7%, P = 0.014). Prevalence of smoking was not different between the two groups (12.9% vs 10.4%, P = 0.171).Prevalence of CAC>0 was 84% in those with DM versus 72.5% in non-DM subjects. Mean CAC score was 343 in DM subjects versus 89 in non-DM subjects.

**Table 1:** Baseline risk factor distributions by groups.

	Total	DM	Non-DM	P
	(n = 1722,100.0%)	(n = 861, 50.0%)	(n = 861, 50.0%)	
**Age, year**	62.1±12.5	62.1±12.5	62.1±12.5	0.9815
**Age≤40 years, n (%)**	87(5.1)	45(5.2)	42(4.9)	0.7413
**Gender, n (%)**				1.000
Male	1084(63.0)	542(63.0)	542(63.0)	
Female	638(37.0)	319(37.0)	319(37.0)	
**Race, n (%)**				1.000
White	742(43.1)	371(43.1)	371(43.1)	
Hispanic	432(25.1)	216(25.1)	216(25.1)	
Black	134(7.8)	67(7.8)	67(7.8)	
Asian	308(17.9)	154(17.9)	154(17.9)	
Others	106(6.2)	53(6.2)	53(6.2)	
**BMI, kg/m** ^**2**^	27.4±7.6	29.9±7.9	26.0±7.1	< .0001**
**Family History of CAD, n (%)**	894(58.7)	451(66.4)	443(52.6)	< .0001**
**Chest Pain, n (%)**	124(28.6)	53(36.1)	71(24.7)	0.014*
**Hyperlipidemia, n (%)**	960(64.4)	522(76.2)	438(54.3)	< .0001**
**Hypertension, n (%)**	914(59.1)	556(77.9)	358(43.0)	< .0001**
**Current smoker, n (%)**	151(11.4)	66(12.9)	85(10.4)	0.171
**Prevalence of CAC (CAC>0), n (%)**	1347(78.2)	723(84.0)	624(72.5)	< .0001**
**CAC Score**	186.5 (3.0–952.0)	343.0 (18.0–1215.0)	89.0 (0.0–653.0)	< .0001**
**CAC Score (CAC>0)**	447.0 (75.0–1220.0)	571.0 (116.0–1510.0)	321.0 (56.5–956.0)	< .0001**
**SIScore**	3.0 (1.0–7.0)	6.0 (3.0–8.5)	2.0 (0.0–5.0)	< .0001**
**TPScore**	4.0 (1.0–10.0)	8.0 (3.0–15.0)	3.0 (0.0–7.0)	< .0001**
**SSScore**	4.0 (1.0–10.0)	9.0 (3.0–17.0)	3.0 (0.0–7.0)	< .0001**

CAC = coronary artery calcium. Data are expressed as mean ± standard deviation, as number (percentage), or as median (inter-quartile range). There were 389 patients with SIScore, TPScore, or SSScore.

*p<0.05

**p<0.001 across groups

**Table 2:** Baseline risk factor distributions for patients with semi-quantitative scores.

	Total
	(n = 389)
**Age, year**	62.9±11.1
**Age≤40 years, n (%)**	11(2.8)
**Gender, n (%)**	
Male	189(48.6)
Female	200(51.4)
**Race, n (%)**	
White	144(37.0)
Hispanic	128(32.9)
Black	37(9.5)
Asian	67(17.2)
Others	13(3.3)
**BMI, kg/m** ^**2**^	27.5±8.0
**Family History, n (%)**	181(47.8)
**Chest Pain, n (%)**	120(31.3)
**Hyperlipidemia, n (%)**	196(53.0)
**Hypertension, n (%)**	200(52.5)
**Current smoker, n (%)**	37(10.1)
**Prevalence of CAC (CAC>0), n (%)**	276(71.0)
**CAC Score**	64.0 (0.0–495.0)
**CAC Score (CAC>0)**	215.5 (40.0–652.0)
**SIScore**	3.0 (1.0–7.0)
**TPScore**	4.0 (1.0–10.0)
**SSScore**	4.0 (1.0–10.0)

CAC = coronary artery calcium. Data are expressed as mean ± standard deviation, as number (percentage), or as median (inter-quartile range).

After adjustmentfor BMI, family history of CAD, history of chest pain, hyperlipidemia, HTN and smoking, among 389 subjects who underwent CTA, SIS (β(Se) = 1.7 (0.3), P <0.001), SSS (β(Se) = 4.6 (0.8), p<0.001) and TPS (β(Se) = 3.7 (0.7), P<0.001) were significantly higher in those with DM (P<0.0001) compared to those without DM. Number of mixed and calcified plaques were significantly higher in those with DM (P = 0.018 and P<0.001 respectively) but there was no significant difference in the number of non-calcified plaques between the two groups (P = 0.398) (Table 3).

**Table 3:** Multiple regression analysis between number of plaques and DM (N = 389).

	Unadjusted Model			Adjusted Model		
	β(Se)	95% C.I.	P	β(Se)	95% C.I.	P
**Number of Non-Calcified Plaques**	0.0 (0.1)	-0.3,0.2	0.794	-0.1 (0.1)	-0.4,0.2	0.398
**Number of Mixed Plaque**	0.6 (0.1)	0.3,0.8	<0.001**	0.3 (0.1)	0.1,0.6	0.018*
**Number of Calcified Plaque**	1.1 (0.2)	0.7,1.4	<0.001**	0.8 (0.2)	0.4,1.2	<0.001**
**SIScore**	2.5 (0.3)	1.8,3.2	<0.001**	1.7 (0.3)	1.1,2.4	<0.001**
**TPScore**	5.0 (0.7)	3.7,6.3	<0.001**	3.7 (0.7)	2.4,5.0	<0.001**
**SSScore**	6.1 (0.7)	4.6,7.5	<0.001**	4.6 (0.8)	3.1,6.2	<0.001**

Adjusted by BMI, family history of CAD, history of chest pain, hyperlipidemia, HTN, smoking status.

*p<0.05

**p<0.001

The number of subjects 40 years old or younger undergoing CTA was very small (11 cases, 2.8%) making the analysis on this specific group impossible because of lack of power. Prevalence of calcified and mixed plaques did increase with age unlike the prevalence of non-calcified plaque which decreased with increased age. All plaque scores were significantly increasing with increasing age (Table 4). CAC score was negatively associated with number of non-calcified plaques but was positively correlated with number of calcified plaques and also all plaque scores (SIS, TPS, TSS) (Table 4).Among patients with DM who were referred by their primary care providers to have cardiac CT in our lab for different reasons, we found that the number of mixed and calcified coronary plaques was significantly higher compared to the age, gender and ethnicity matched individuals who did not have DM.

**Table 4:** Correlation coefficients of semi-quantitative scores with coronary artery calcium scores or age.

	CAC	Age
**SIScore**	0.50**	0.34**
**TPScore**	0.66**	0.33**
**SSScore**	0.60**	0.30**
**Number of Non-Calcified Plaques**	-0.20**	-0.04
**Number of Mixed Plaque**	0.18**	0.13*
**Number of Calcified Plaque**	0.55**	0.35**

*p<0.05

**p<0.001

The fact that our DM population had significantly higher report of chest pain in their history even though DM is known to blunt chest pain as a presentation of CAD[26], indicates that they likely had more advanced CAD in comparison to their not-DM counterparts. This was reflected by higher CAC score and also higher prevalence of mixed plaque in our DM patients. Higher CAC score and also higher prevalence of mixed plaques have been both associated with poorer outcome of CVD[27]. These findings signifies the necessity of preventive measures to screen CAD in early stages in patients with DM in order to implement more intense risk factor modifications to prevent progression of CAD in this population.

Prevention requires effective diagnostic techniques to identify people at riskbefore the onset of severe clinical events. Assessment of reversal and prevention requires application ofnoninvasive procedures that accurately quantify the extent of coronary atherosclerosis. CAC measured by cardiac CT is noninvasive, and have been shown to correlate with asymptomatic and symptomatic CAD. A large numberof longitudinal studies have now demonstrated a strong predictive relationship between the extent of CACand the frequency of subsequent clinical events [28, 29]. This predictive relationship is present among bothmen and women and across all ethnicities. The ACCF/AHA guidelines for assessment ofcardiovascular risk in asymptomatic adults contain for the first time a Class IIa indication for CACscanning in asymptomatic patients with DM[30]. In previous studies, the measured CAC in persons withDM without known CAD was similar to that found in adults without DM with known CAD [2, 4, 31].

On the other hand as calcificationmay be a late finding in the pathogenesis of atherosclerosis, it may underestimate CAD, especially in ayounger DM population and it has been demonstrated that there is not a linear relationship between CAC and overall plaque burden; consequently CAC may not be a sufficient tool to determine overallplaque burden[32, 33].

The assessment of mixed plaques by CT-angiography may allow for improved cardiovascularrisk stratification and determine the presence of early atherosclerosis in patients with DM.

Unfortunately the number of patients with DM who were younger than 40 in our studywas not enough to provide us with statistical power for further comparison of CAC score and semiquantitave plaque scores to those without DM.

CAC data on young persons with DM is very limited. Starkman et al measured CAC in young adults with DM1 of for at least 5 years and found that 10.9% had CAC [34]. Hoff et al found that younger individuals with DM had CAC comparable to older adults without DM, but the population with patients aged <40 were few [35].

## Conclusions

Our findings of higher CAC score and higher prevalence of mixed plaque in patients with DM versus those without DM reinforces the benefits of CAC scoring and CTA in detection of CAD in DM subjects. However the fact that our study subjects were referred for further evaluation for CAD which will introduce bias by itself; and also the small number of cases younger than 40 and lack of control group;warrants further studies on asymptomatic DM patients of different types with focus on younger ages and with inclusion of a healthy control group. This can provide meaningful data for formulating preventive strategies in individual with DM and to decrease their CVD morbidity and mortality. This could inform future screening and treatment of CAD in DM, and thus will be of high clinical impact and will be the key for developing new strategies to reduce CAD-related morbidity and mortality among persons with DM including younger patients.

The strength of our study is the large number of cases with DM with available semi-quantitative scores reported from one center. We have previously showed a high degree of inter-observer agreement in the measurement of plaque amount [23] and reproducibility of our reports[36].

There are also some limitations to our study. First, we did not know subtypes of DM in our population and the epidemiology of CAD and also associated CVD risk factors might be different in DM1 versus DM2. Second, we did not have a control group to compare our findings to a healthy asymptomatic population.
